# Modeling and simulation of maintenance treatment in first-line non-small cell lung cancer with external validation

**DOI:** 10.1186/s12885-016-2455-2

**Published:** 2016-07-13

**Authors:** Kelong Han, Laurent Claret, Alan Sandler, Asha Das, Jin Jin, Rene Bruno

**Affiliations:** GlaxoSmithKline, Clinical Pharmacology Modeling & Simulations, 709 Swedeland Rd, King of Prussia, PA 19406 USA; Genentech/Roche, 84 Chemin des Grives, 13013 Marseille, France; Genentech Inc, Product Development Oncology, South San Francisco, CA USA; Tocagen Inc, Clinical Development and Medical Affairs, San Diego, CA USA; Genentech Inc, Clinical Pharmacology, South San Francisco, CA USA

**Keywords:** Non-small cell lung cancer, Maintenance treatment, Tumor growth inhibition, Trial simulation, Overall survival, External validation

## Abstract

**Background:**

Maintenance treatment (MTx) in responders following first-line treatment has been investigated and practiced for many cancers. Modeling and simulation may support interpretation of interim data and development decisions. We aimed to develop a modeling framework to simulate overall survival (OS) for MTx in NSCLC using tumor growth inhibition (TGI) data.

**Methods:**

TGI metrics were estimated using longitudinal tumor size data from two Phase III first-line NSCLC studies evaluating bevacizumab and erlotinib as MTx in 1632 patients. Baseline prognostic factors and TGI metric estimates were assessed in multivariate parametric models to predict OS. The OS model was externally validated by simulating a third independent NSCLC study (*n* = 253) based on interim TGI data (up to progression-free survival database lock). The third study evaluated pemetrexed + bevacizumab vs. bevacizumab alone as MTx.

**Results:**

Time-to-tumor-growth (TTG) was the best TGI metric to predict OS. TTG, baseline tumor size, ECOG score, Asian ethnicity, age, and gender were significant covariates in the final OS model. The OS model was qualified by simulating OS distributions and hazard ratios (HR) in the two studies used for model-building. Simulations of the third independent study based on interim TGI data showed that pemetrexed + bevacizumab MTx was unlikely to significantly prolong OS vs. bevacizumab alone given the current sample size (predicted HR: 0.81; 95 % prediction interval: 0.59–1.09). Predicted median OS was 17.3 months and 14.7 months in both arms, respectively. These simulations are consistent with the results of the final OS analysis published 2 years later (observed HR: 0.87; 95 % confidence interval: 0.63–1.21). Final observed median OS was 17.1 months and 13.2 months in both arms, respectively, consistent with our predictions.

**Conclusions:**

A robust TGI-OS model was developed for MTx in NSCLC. TTG captures treatment effect. The model successfully predicted the OS outcomes of an independent study based on interim TGI data and thus may facilitate trial simulation and interpretation of interim data. The model was built based on erlotinib data and externally validated using pemetrexed data, suggesting that TGI-OS models may be treatment-independent. The results supported the use of longitudinal tumor size and TTG as endpoints in early clinical oncology studies.

**Electronic supplementary material:**

The online version of this article (doi:10.1186/s12885-016-2455-2) contains supplementary material, which is available to authorized users.

## Background

There is still an unmet medical need in the treatment of non-small cell lung cancer (NSCLC) in both the first-line and recurrent settings. Maintenance treatment has been investigated in patients with disease control (i.e. without progressive disease) during first-line therapy in a number of trials with the goal to prolong time to disease progression (progression-free survival, PFS), improve quality of life and ultimately prolong overall survival (OS) [1–4]. However, the risk-benefit ratio of maintenance therapy in NSCLC is still unclear, and several aspects of this strategy have raised considerable debate [2]. Therefore models that could predict the clinical outcomes of maintenance therapy may be of great importance to practitioners and drug developers.

Modeling and simulation may provide quantitative support for interpretation of interim data and development decisions in oncology [5, 6]. Tumor response of patients can be characterized using tumor growth inhibition (TGI) metrics, which are estimated based on modeling of longitudinal tumor size data. TGI metrics have been shown to predict treatment effect on OS in solid tumors and in multiple myeloma [5]. These TGI metrics include model-based estimates of change in tumor size from baseline at end of cycle 2 (e.g. week 6 or 8), tumor growth rate and time to tumor regrowth [5]. TGI metrics could be used as alternative endpoints [7] in early clinical studies to optimize drug dosing, support clinical trial design for investigational anti-cancer treatments [5, 6].

Although a few models linking OS with TGI metrics and prognostic factors have been published for NSCLC first-line [8–10] and second-line [8] therapies, there has been no investigation of TGI metrics and of their link to OS in the context of maintenance therapy to date. Furthermore, there is insufficient published external validation of such models. External validation is critical for assessing treatment independence of the models and favour acceptance [5]. Finally, the OS models are assumed to be disease-specific but treatment-independent. However, to date, there has been insufficient validation of the treatment-independence assumption.

Accumulation of valuable clinical data has made it possible to build and externally validate a TGI-OS model for maintenance therapy in NSCLC patients whose disease did not progress during first-line therapy. Erlotinib maintenance prolonged both PFS [11] and OS [12] in the SATURN trial. The addition of erlotinib to bevacizumab during maintenance therapy significantly prolonged PFS but not OS compared to the bevacizumab-only maintenance in the ATLAS trial [13]. The AVAPERL trial compared maintenance bevacizumab plus pemetrexed vs. bevacizumab alone and showed a significant prolongation of PFS [14] but not of OS [15] following bevacizumab plus pemetrexed compared to bevacizumab alone.

The objectives of this work were 1) to develop a model for OS after maintenance therapy in NSCLC based on erlotinib data from SATURN and ATLAS, 2) to prospectively predict the probability to success of AVAPERL study and perform an external validation by simulating the OS outcomes of AVAPERL study (pemetrexed data) based on interim tumor size data (up to PFS database lock).

## Methods

### Trials and data

Data were collected from all patients enrolled in three studies evaluating maintenance treatment after first-line NSCLC therapy. In all studies, patients whose disease did not progress after four cycles of first-line treatment were randomized to maintenance treatment. Details of the studies can be found in the respective papers, in the introduction section and in Table 1. The studies complied with the Declaration of Helsinki and Good Clinical Practice guidelines, and were approved at all investigating centers by local ethics committees. All patients provided written informed consent for participation and publication of the data [11–15]. An ethics statement was not required for this analysis as they have been provided in each of the three individual studies [11–15].

**Table 1:** Study summary

	SATURN [11, 12]	ATLAS [13]	AVAPERL [14, 15]
Investigational drug	Erlotinib	Erlotinib	Pemetrexed
N: run-in phase^a^	1949	1145	376
N: maintenance phase^b^	889	743	253
N: evaluable^c^	837 (94 %)	697 (94 %)	231 (94 %)
BTS (cm)	6.99	6.1	5.21
Female^d^	26 %	48 %	43 %
ECOG score >0^d^	69 %	66 %	52 %
Age ≥ 55 years^d^	70 %	77 %	72 %
Asian^d^	15 %	13 %	12 %
Study number	BO18192	AVF3671g	MO22089
ClinicalTrials.gov Identifier	NCT00556712	NCT00257608	NCT00961415
Trial registration date^e^	Nov 9, 2007	Nov 21, 2005	Aug 18, 2009
Retrospective registration	No	No	No

*BTS* baseline tumor size at randomization, *ECOG* Eastern Cooperative Oncology Group, *TGI* tumor growth inhibition

^a^Number of patients who received four cycles of first-line treatment (run-in phase)

^b^Number of patients whose disease did not progress during the run-in phase and who were randomized in the maintenance phase

^c^Number of evaluable patients, i.e. patients with at least two tumor size measurements in the maintenance phase (at least one tumor size measurement after randomization). The number in the parenthesis represents the percentage of evaluable patients out of the patients randomized

^d^The percentage of patients among evaluable patients

^e^The date of “First received” as displayed on ClinicalTrials.gov

The SATURN trial compared maintenance erlotinib vs. placebo in patients whose disease did not progress after four cycles of platinum-based first-line chemotherapy [11, 12]. The ATLAS trial compared maintenance erlotinib plus bevacizumab vs. bevacizumab alone in patients whose disease did not progress after four cycles of platinum-doublet chemotherapy in combination with bevacizumab [13]. The AVAPERL trial compared maintenance bevacizumab plus pemetrexed vs. bevacizumab alone in patients whose disease did not progress after four cycles of first-line chemotherapy of cisplatin plus pemetrexed in combination with bevacizumab [14, 15].

The following baseline patient characteristics were tested as prognostic factors for OS based on SATURN and ATLAS data: age, gender, ethnicity, Eastern Cooperative Oncology Group (ECOG) score, smoking status, tumor size, and histology. In addition, study effects and response to first-line therapy were investigated. Interim AVAPERL data consisted in longitudinal tumor size collected by the time of PFS database lock (data cutoff: July 2011) and baseline patient characteristics only.

### Tumor growth inhibition metrics

**Fig. 1:**
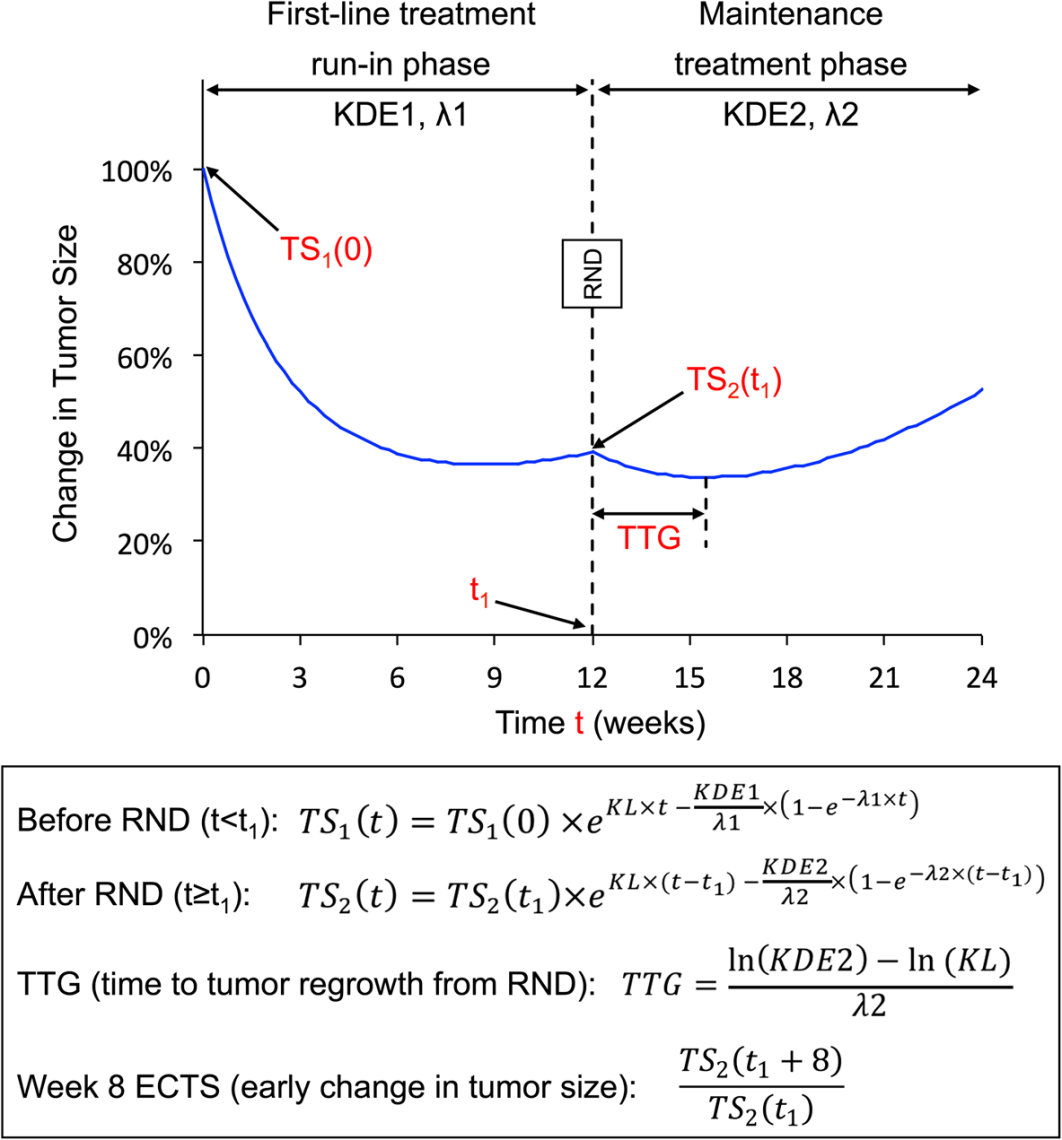
Theoretical tumor size profile over first-line treatment run-in phase and maintenance treatment phase. t_1_: time of randomization to maintenance treatment; KL: growth rate (assumed to be same during the two treatment phases); TS, KDE and λ: tumor size, drug effect and drug effect decay over time, respectively, for the first-line (TS_1_, KDE1 and λ1) and maintenance (TS_2_, KDE2 and λ2) phase; RND: randomization

The full TGI profile was modeled using equations adapted from previously published simplified TGI models [16] (Fig. 1) that were fit to data from evaluable patients using a nonlinear mixed-effect modeling (population) approach (NONMEM, version 7, FOCE algorithm with interaction) [17]. To be evaluable in this analysis, patients had to have at least one tumor size measurement after randomization to maintenance treatment. Tumor size was assessed as the sum of longest diameters of target lesions by Response Evaluation Criteria In Solid Tumors (RECIST) [18, 19]. Shrinkage in model-parameter estimates was estimated as previously described [20]. Model fitting was assessed using standard goodness-of-fit plots.

Two patient-level TGI metrics were calculated based on individual posthoc parameter estimates: the time to tumor regrowth (TTG) [16], and the week 8 ECTS (early change in tumor size) that represented early tumor shrinkage and was calculated as the ratio of model-predicted tumor size at week 8 to baseline estimated by the model. Equations are displayed in Fig. 1. Only the TGI metrics during the maintenance phase were of interest and were calculated.

### Overall survival model development

Data from SATURN and ATLAS were used to build the OS model. The impact of individual factors on OS was assessed using Kaplan-Meier and Cox regression analyses using *survfit* and *coxph* functions, respectively in R (version 2.15.0) [21]. The baseline patient prognostic factors together with the TGI metrics were tested to explain variability in OS.

A parametric survival regression model (using the *survreg* function in R version 2.15.0) was developed that describes OS distribution. The probability density function that best describes the observed survival time was selected among normal, lognormal, Weibull, logistic, log-logistic, and exponential by using difference in Akaike information criterion (AIC) [22] of the alternative models.

A “full” model was built by including all significant covariates (baseline prognostic factors, TGI metrics) from the Cox univariate analysis with a significance level of *p* < 0.05 per the log-likelihood ratio test where the difference in −2*log-likelihood (score) between alternative models follows a χ^2^ distribution. The score indicates the level of significance for the association between this covariate and OS: the higher the score, the more significantly this covariate is associated with OS. Then a backward stepwise elimination was carried out. At each elimination step, one covariate was removed from the model. If the reduced model (without this removed covariate) became significantly worse (*p* < 0.01), the removed covariate stayed in the model. The relative influence of each remaining covariate on the model was re-evaluated by deleting it from the reduced model on an individual basis with a significance level of *p* < 0.01. The backward elimination resulted in the final model, in which all covariates were significant.

The model simulation performances were evaluated using a posterior predictive check. OS distributions and hazard ratios (HR) in SATURN and ATLAS were simulated 1000 times. Model parameters were sampled from the estimated mean values and uncertainty in parameter estimates for each of the simulated study replicate. Censoring was assumed to be 30 % as in the original data.

### Simulations

OS of AVAPERL study were simulated based on TGI metrics estimated using interim tumor size data to predict the likelihood of a successful OS outcome for AVAPERL and further assess performance of the OS model (external validation). In order to calculate the prediction interval and make statistical inferences, the study was simulated multiple times (20,000) by sampling survival model parameters from their estimated uncertainty distribution. Patient survival times were drawn from the appropriate survival distribution defined by model parameters, baseline prognostic factors and TGI metric of AVAPERL patients. Censoring was simulated in sampling patient study duration, assumed to be independent of death. Patient survival times were censored assuming a uniform distribution of patient study duration from 50 to 140 weeks, which was consistent with the minimum and the maximum time period the patient stayed in the SATURN study without a death event. For each of the replicates, simulated data were analyzed by Kaplan-Meier estimation and Cox regression. Kaplan-Meier estimates of OS distributions and HR used to compare both arms were summarized by median and 95 % prediction interval (PI) across the replicates.

## Results

### Data

Patients with at least one post-randomization tumor size measurement were included in this analysis. Overall 1534 patients were evaluable to estimate TGI metrics used for building the OS model: 837 (94 %) out of 889 patients from SATURN, and 697 (94 %) out of 743 patients from ATLAS. Interim AVAPERL data used as the external validation dataset were collected by the time of PFS database lock (data cutoff: July 2011) and included 231 evaluable patients out of 245 (94 %) randomized to maintenance treatment.

### Tumor size model

The simplified TGI model adequately described the observed tumor size data, as shown by goodness-of-fit plots and individual fits (Additional file 1: Figure S1 and Additional file 2: Figures S2). Parameters were adequately estimated with small standard errors and shrinkage (Table 2) except that inter-individual variability could not be estimated on λ1 due to the limited number of observations during first-line treatment phase. TGI metric estimates (TTG and week 8 ECTS) that were calculated from the TGI model parameters (Table 2) using equations displayed in Fig. 1 were highly variable: the range from 5th to 95th percentile was 0.721 (i.e. decrease in tumor size from baseline) to 1.24 (i.e. increase in tumor size from baseline) for week 8 ECTS, and −23 weeks to 70 weeks for TTG after randomization. TTG may take negative values when KL > KDE2, i.e. in patients with progression at the first assessment after randomization to maintenance phase (Additional file 2: Figure S2).

**Table 2:** Parameter estimates of the simplified TGI model

	Estimate	RSE (%)	IIV	Shrinkage (%)
KL (week^−1^)	0.00464	8.59	1.05	25.9
KDE1 (week^−1^)	0.0566	3.95	0.699	18.3
λ1 (week^−1^)	0.117	6.06	Fixed to 0	
KDE2 (week^−1^)	0.00412	18.2	1.64	42.1
λ2 (week^−1^)	0.0597	14.9	0.787	74.3
BASE (cm)	7.74	1.67	0.642	3.5
σ^2^ (cm^2^)	0.58	9.14	-	28.8

*BASE* estimated baseline tumor size, *IIV* standard deviation of inter-individual variability, *KDE and λ* drug effect and drug effect decay over time, respectively for first-line treatment run-in phase (KDE1 and λ1) and maintenance treatment phase (KDE2 and λ2), *KL* growth rate (assumed to be same during the two treatment phases), *RSE* relative standard error of parameter estimates, *TGI* tumor growth inhibition, *σ* standard deviation of residual variability

### Overall survival model

**Fig. 2:**
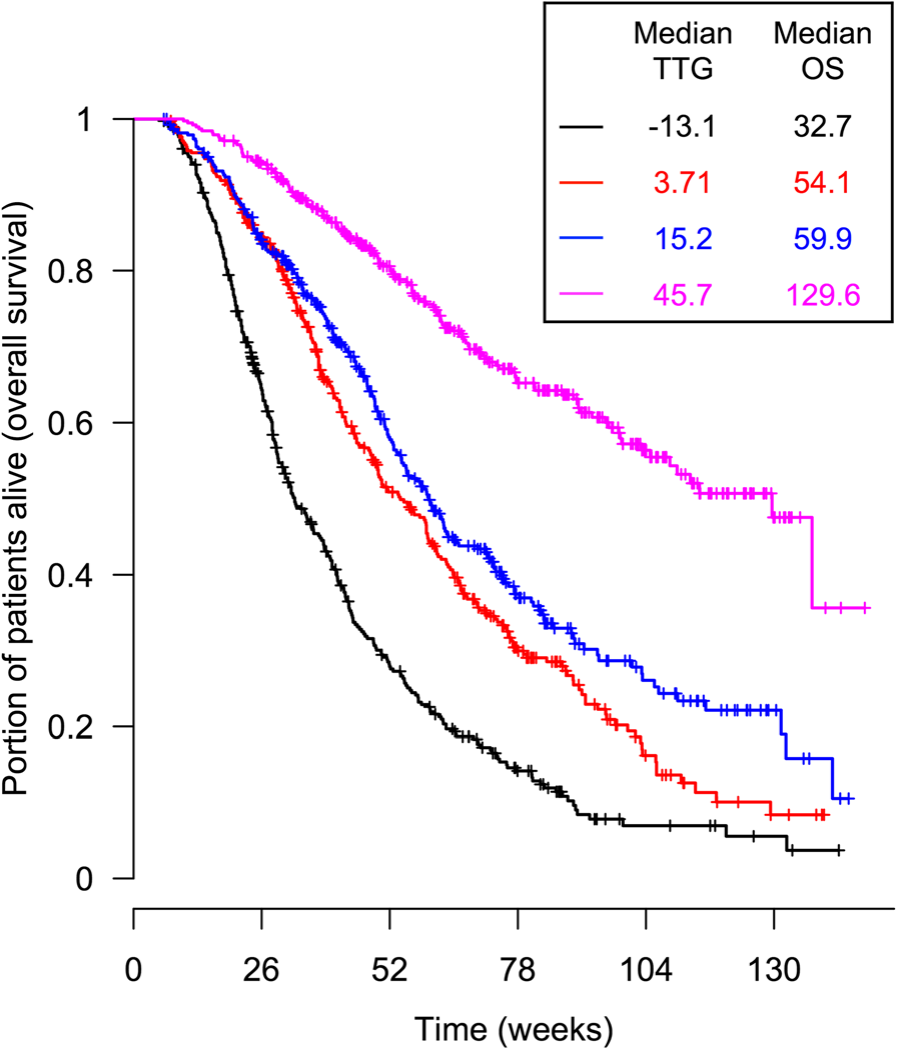
Overall survival by quartiles of TTG. Each group represents 25 % of the patients. TTG: time to tumor regrowth (week). OS: overall survival (week). Median estimates are reported in the insert

In univariate Cox analysis (Table 3), TTG was the most significant covariate associated with OS (score 151.7) and much better than week 8 ECTS (score 45.1). The most significant baseline prognostic factors and patient characteristics were tumor size, gender, smoking status, Asian ethnicity and ECOG score (scores 8 to 50, *p* < 0.0001). Also OS tended to be longer in erlotinib treated patients and in ATLAS trial compared to SATURN (*p* < 0.01). OS distribution by quartiles of TTG is shown in Fig. 2.

**Table 3:** Screening of the potential covariates for overall survival using the Cox model

	HR	95 % CI	Score	*p*	Sign on risk
TTG (weeks)	0.83^a^	0.81–0.85^a^	151.7	<0.0001	−
Tumor size at randomization (cm)	1.17^b^	1.13–1.20^b^	51.8	<0.0001	+
Week 8 ECTS	1.12^c^	1.10–1.14^c^	45.1	<0.0001	+
Female	0.64	0.56–0.74	21.2	<0.0001	−
Never smoked	0.57	0.47–0.68	20.3	<0.0001	−
Asian	0.61	0.50–0.75	12.5	<0.0001	−
Study SATURN	1.33	1.16–1.51	9.2	<0.0001	+
ECOG score >0	1.30	1.14–1.49	7.7	0.0001	+
Age ≥ 55 years	1.23	1.07–1.42	4.2	0.0037	+
Squamous	1.22	1.06–1.40	3.8	0.0060	+
Erlotinib	0.85	0.75–0.96	3.5	0.0082	−
Erlotinib in SD	0.84	0.73–0.97	3	0.0144	−
Age (years)	1.07^d^	1.01–1.14^d^	2.6	0.0221	+

*CI* confidence interval, *ECTS* early change in tumor size, *Erlotinib* patients who received erlotinib during the first-line treatment run-in phase (all patients were analyzed), *Erlotinib in SD* patients who received erlotinib during the first-line treatment phase (only those patients who achieved stable disease during first-line treatment run-in phase were analyzed), *HR* hazard ratio, *p* obtained by likelihood ratio test, *Score* log(likelihood ratio), *Sign on risk* + (−) indicates that this variable is associated with increased (decreased) risk, *TTG* time to tumor regrowth, ^a^HR for increase of every 8 weeks of TTG; ^b^HR for increase of every 2 cm of tumor size at randomization; ^c^HR for increase in every 10 % of tumor size as compared to the randomization; ^d^HR for increase of every 10 years of age

A lognormal distribution had the best likelihood to describe the OS distribution (lower AIC than other distributions). All covariates that were significant in the Cox univariate analysis were included in the “full” model, and underwent backward stepwise elimination. The final model included TTG and the following baseline prognostic factors: baseline tumor size, ECOG score (0 vs. >0), Asian ethnicity, age and gender. All parameters in the final OS model were estimated with good precision (Table 4). According to the model, good prognostic is predicted for patients with longer TTG (treatment effect), small baseline tumor size, age below 55 years, Asian ethnicity, ECOG score 0 and for female patients.

**Table 4:** Parameter estimates of the final overall survival model

	Estimates	Standard Error	*p*
(Intercept)	4.3776	0.065883	<0.00001
TTG (weeks)	0.0139	0.000889	<0.00001
BTS (cm)	−0.0437	0.005014	<0.00001
Age ≥ 55 years	−0.2519	0.049494	<0.00001
Asian	0.2324	0.066116	0.00044
ECOG score >0	−0.157	0.045344	0.00054
Female	0.1437	0.045306	0.00151
Log(scale)	−0.3017	0.024079	<0.00001

Overall survival was modeled in weeks. A positive (negative) value of the estimate indicates that an increase (decrease) in the value of this variable is associated with favorable (unfavorable) overall survival outcome. The *p* value was obtained by Wald test (χ^2^). *BTS* baseline tumor size at randomization, *TTG* time to tumor regrowth

**Fig. 3:**
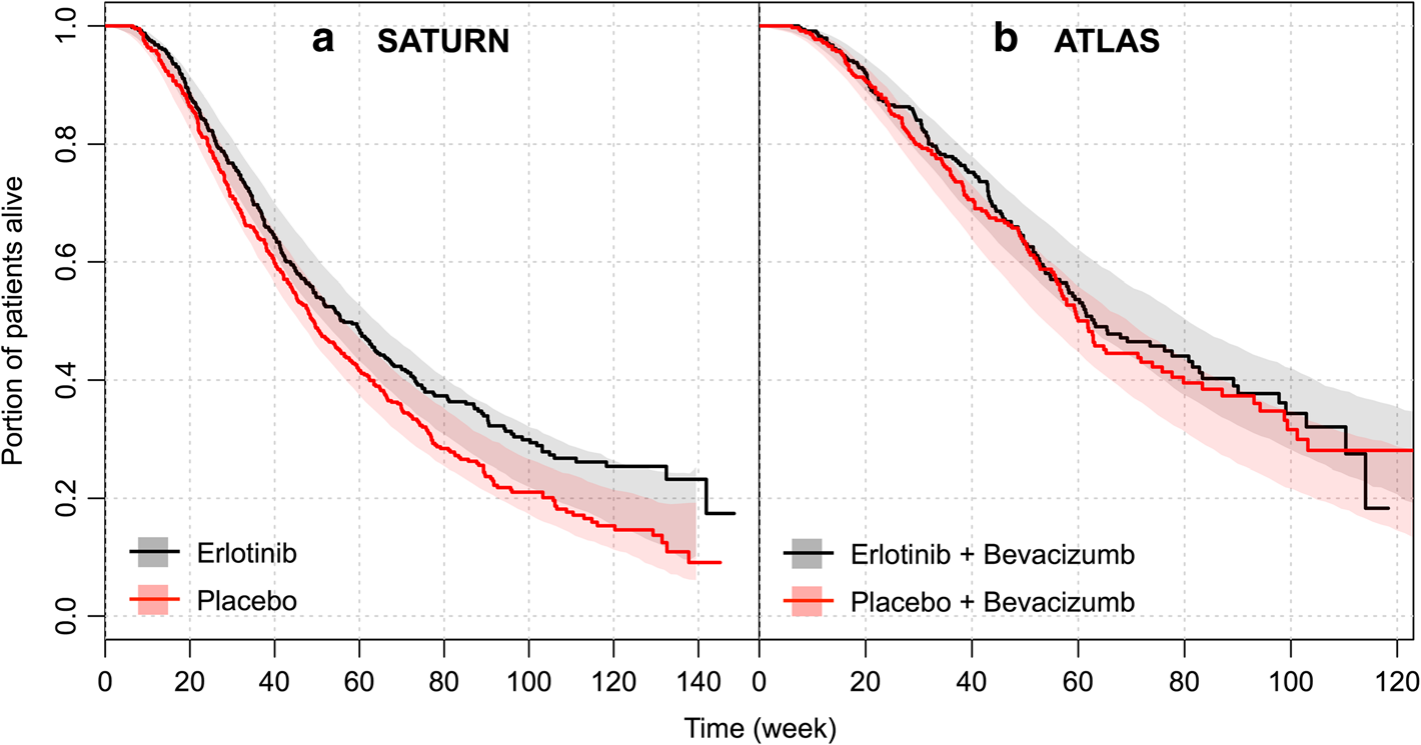
Posterior predictive check of the final OS model by studies: **a**) SATURN and **b**) ATLAS. Solid line: observed OS. Band: 95 % prediction interval of OS. OS: overall survival

**Fig. 4:**
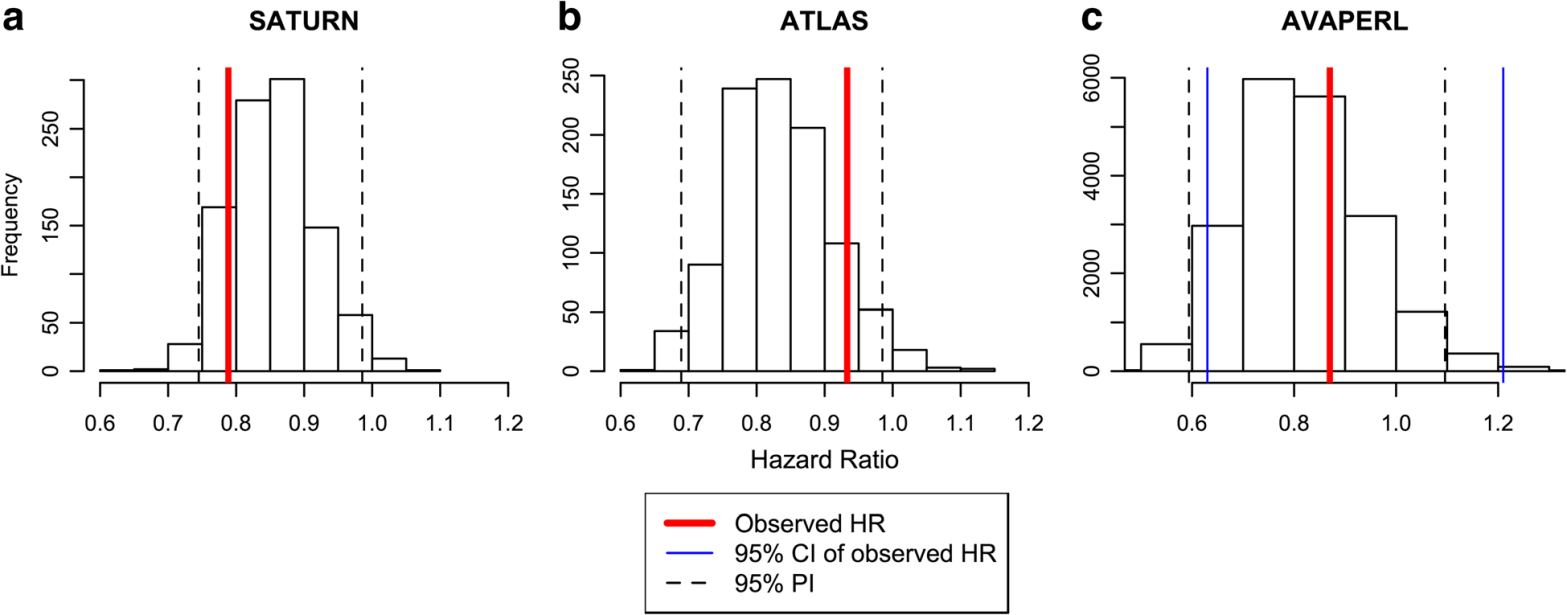
Posterior predictive check of HR in SATURN (**a**) and ATLAS (**b**) for maintenance erlotinib compared to placebo and simulation of HR in AVAPERL (**c**) for maintenance pemetrexed vs. placebo and comparison to observed HR. CI: confidence interval. HR: hazard ratio. PI: prediction interval

The model was evaluated by simulating OS distributions in each of the study arms (Fig. 3) and the HR of treatment vs. control arm in SATURN and ATLAS (Fig. 4a and b). The observed HR (0.79 for SATURN and 0.93 for ATLAS) was within the 95 % PI by the model (0.74–0.97 for SATURN and 0.70–1.00 for ATLAS).

### Simulation

The final OS model was applied to prospectively predict the expected OS outcome of AVAPERL study (external validation). The goal was to predict the likelihood of a successful OS outcome using interim tumor size data collected by the time of PFS database lock (data cutoff: July 2011). This dataset was not used for model-building (Table 1). Median OS was not yet reached at the time of data cutoff, and the immature OS data that were observed by the time of data cutoff were not used. Patients in AVAPERL study had more favorable prognostic factors than those from SATURN and ATLAS with a smaller proportion of ECOG score >0 (52 % vs. 66–69 %) and smaller baseline tumor size (5.2 cm vs. >6 cm) (Table 1). Simulations indicated that pemetrexed plus bevacizumab as maintenance treatment in AVAPERL was unlikely to demonstrate a significant OS prolongation vs. bevacizumab alone. The expected HR was 0.81 with a 95 % PI of 0.59–1.09 (62 % of events), which contained 1 (Fig. 4c). Predicted median OS was 17.3 and 14.7 months in both arms, respectively. These prospective simulations were consistent with the results of the final OS analysis published recently [15]: the final observed HR was 0.87 with a 95 % confidence interval of 0.63–1.21 (58 % of events). The final observed median OS was 17.1 and 13.2 months in both arms, respectively.

## Discussion

Maintenance treatment in responders after induction first-line treatment, without waiting for disease progression and start of a new line of therapy, is a therapeutic strategy investigated and used in several tumor types including adult and pediatric acute lymphocytic leukemia [23, 24], follicular non-Hodgkin lymphoma [25, 26], multiple myeloma [27], breast cancer [28], metastatic colorectal cancer [29, 30], and advanced ovarian cancer [31–33]. Although well established for certain hematologic cancers, maintenance therapy has only recently become a treatment option for NSCLC [1–3]. The risk-benefit ratio of maintenance therapy in NSCLC is still unclear, and the thoracic oncology community has seen considerable debate over several aspects of this strategy [2]. Even when maintenance treatment allows prolonging PFS and possibly OS, it is unclear whether OS is prolonged compared to classical first-line followed by second-line paradigm. The selection of patients likely to benefit warrants further research [1–3].

Model-based approaches are gaining momentum to optimize anti-cancer drug usage and development [6]. Estimates of TGI metrics from modeling of longitudinal tumor size data have been used to predict clinical outcomes and simulate clinical trials [5] in variety of settings including first- and second-line treatment of NSCLC [8–10]. We present here an adaptation of the modeling framework for maintenance treatment in NSCLC. The framework is developed based on two erlotinib maintenance studies and assessed in simulating outcome of an independent pemetrexed study. As observed in first-line treatment [9, 10], an estimate of time to tumor regrowth (TTG) after start of maintenance treatment captured drug effect, i.e. an OS model incorporating TTG and baseline prognostic factors was able to simulate erlotinib HR in SATURN and ATLAS. Baseline prognostic factors in the model are well known prognostic factors for OS: good prognostic for patients with small baseline tumor size, age below 55 years, Asian ethnicity, ECOG score 0 and for female patients. Smoking status and histology (squamous vs. non-squamous) that were of significant prognosis in the univariate analysis were not retained in the final multivariate model.

As previously discussed [16], the TGI model does not account for exposure to the treatment drugs and is not subjected to any simulation-based assessment (e.g. visual predictive check) because it is not meant to be used for simulation but only to estimate the TGI metrics to be used in the OS model. The TGI model could be in other forms as well, such as a combination of exponential and/or linear models [8, 34] or a simple spline function. Therefore the fundamental assumption of constant exposure over time that was previously used [35] to derive this TGI model from the more complex exposure-driven model is irrelevant here as the model is not used in simulations of response for alternative exposure. There is also no need to assess covariate effects on the TGI model parameters because the model is not used to simulate tumor sizes in new patients.

We performed a two-stage analysis, meaning that we first estimated TGI metrics and then developed the OS model, and we thereby ignored time-dependent hazard driven by time-dependent tumor size. In a typical clinical trial setting, tumor size is only observed until disease progression when treatment is stopped. Median time of last tumor size observation was 11–18 weeks while median OS was four times as long (45–63 weeks) in our model-building dataset. As a result TTG is much shorter than time to death as shown with the median estimates of TTG and OS in Fig. 2. Accounting for tumor size-dependent hazard would have implied an extrapolation substantially beyond last tumor size observation, leading to unrealistically large tumor sizes as the model assumes exponential growth after end of treatment. Information about subsequent treatments is usually unavailable, while tumor size-dependent hazard could only be implemented and evaluated with richer data that could be obtained during routine care of the patients across several lines of treatments when tumor size data could be observed and hazard defined up to patient death. This approach has been explored with PFS, which does not suffer this problem [36]. Additionally, simulations have shown that TTG was not confounded with OS [37, 38].

In the OS model, the censoring model is meant to mimic the duration (treatment plus follow-up period) a patient stays in the study if no death event occurs. The distribution of this duration is defined per protocol by the maximum duration of the study and the patient inclusion rate. If a patient is predicted to die after his predicted duration in the study, this patient is censored. The distribution of study duration is independent of OS and TGI data and doesn’t require simultaneous modeling.

Another limitation of our analysis is that patients needed to have at least two tumor size measurements in the maintenance phase to be evaluable in the TGI model because the TGI parameters were unidentifiable with only one tumor size measurement. These excluded patients who died or dropped out of the study early before the first tumor size measurement may have rapidly growing tumors. However, this may not have a significant impact on this analysis because 94 % of the patients were evaluable.

The model successfully simulated the OS outcomes of the pemetrexed maintenance study AVAPERL based on interim tumor size data collected by the time of PFS database lock before median OS was even reached. This is the first modeling framework for maintenance treatment and one of the few such frameworks validated in simulating an independent study with a drug with a different mechanism of action (pemetrexed) compared to the one used to develop the model (erlotinib), providing support to the hypothesis that TGI metrics capture drug effect independent of treatment [5]. This framework may be used to support design and interim analysis of upcoming maintenance studies and to help in the selection of patients most likely to benefit from maintenance treatment.

## Conclusion

In conclusion, a robust TGI-OS model linking OS with TGI metrics and prognostic factors was developed for maintenance therapy following first-line NSCLC treatment. The model successfully predicted the OS outcomes of an independent study (AVAPERL) based on interim tumor size data (up to PFS database lock), indicating that the model may be used for trial simulation and facilitate interpretation of interim data and development decisions. The model was built based on erlotinib data and externally validated using pemetrexed data, suggesting that TGI-OS models may be treatment-independent. The results also supported the use of longitudinal tumor size and TTG as endpoints in early clinical oncology studies.

## Abbreviations

AIC, Akaike information criterion; ECOG, Eastern Cooperative Oncology Group; ECTS, early change in tumor size; HR, hazard ratio; MTx, maintenance treatment; NSCLC, non-small cell lung cancer; OS, overall survival; PFS, progression-free survival; PI, prediction interval; TGI, tumor growth inhibition; TTG, time to tumor regrowth

## Additional files

Additional file 1: Figure S1. Goodness of fit plot of the simplified tumor growth inhibition (TGI) model. (TIFF 3241 kb) Additional file 2: Figure S2. Model predicted vs. observed tumor size for 16 individuals taken at random. Black solid points: observed tumor size. Orange solid line: model predicted tumor size. Grey dash line: time of randomization (start of maintenance treatment). Grey solid line: estimated time to tumor regrowth (TTG). (TIFF 3041 kb)
