# Supplementary figures and images for: Modeling and simulation of maintenance treatment in first-line non-small cell lung cancer with external validation
Source: BMC Cancer. 2016 Jul 13;16:473. doi: 10.1186/s12885-016-2455-2 (PMC4944249; doi:10.1186/s12885-016-2455-2)

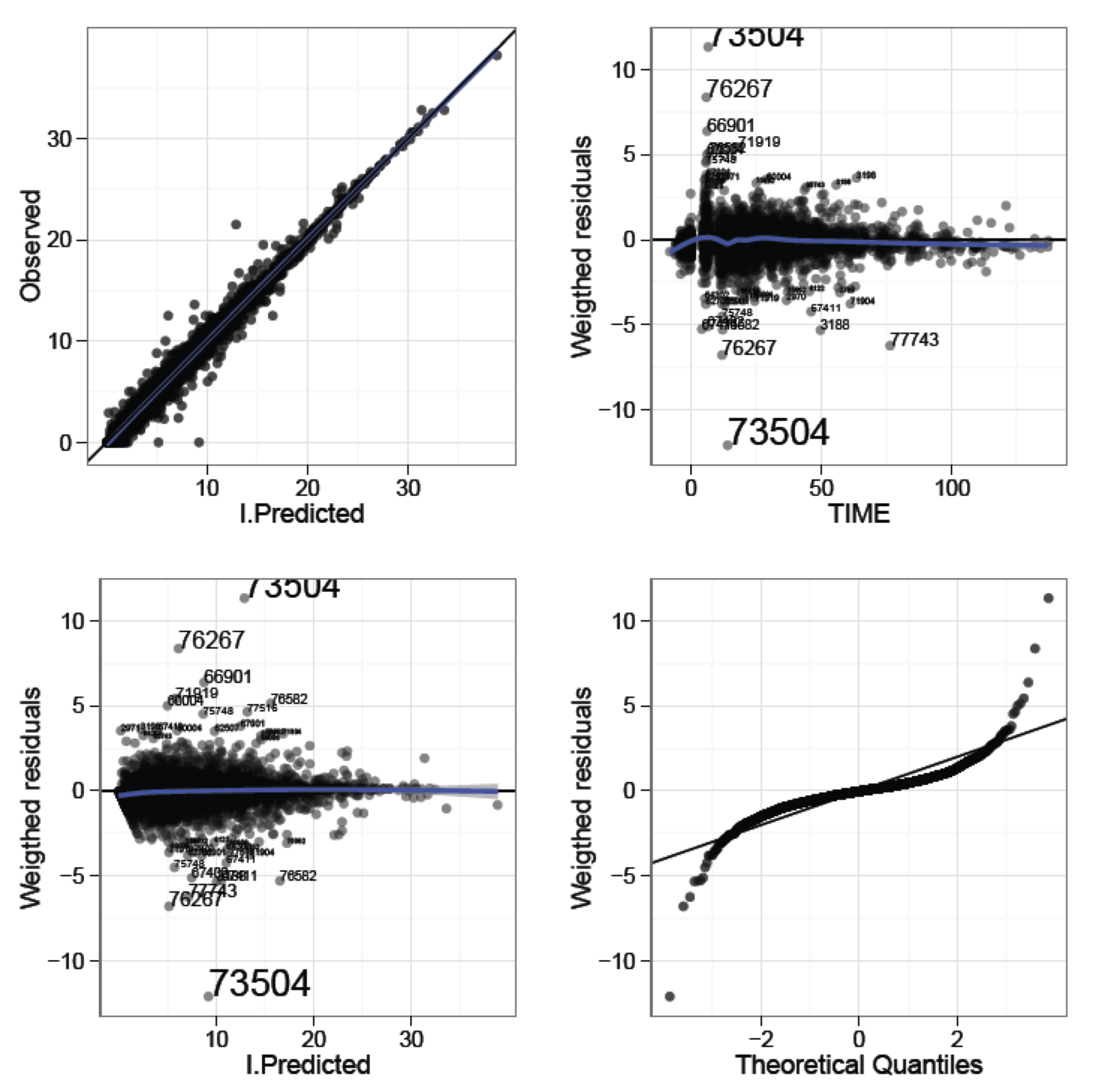

Supplement: Additional file 1: Figure S1. — Goodness of fit plot of the simplified tumor growth inhibition (TGI) model. (TIFF 3241 kb) [file 12885_2016_2455_MOESM1_ESM.tiff]

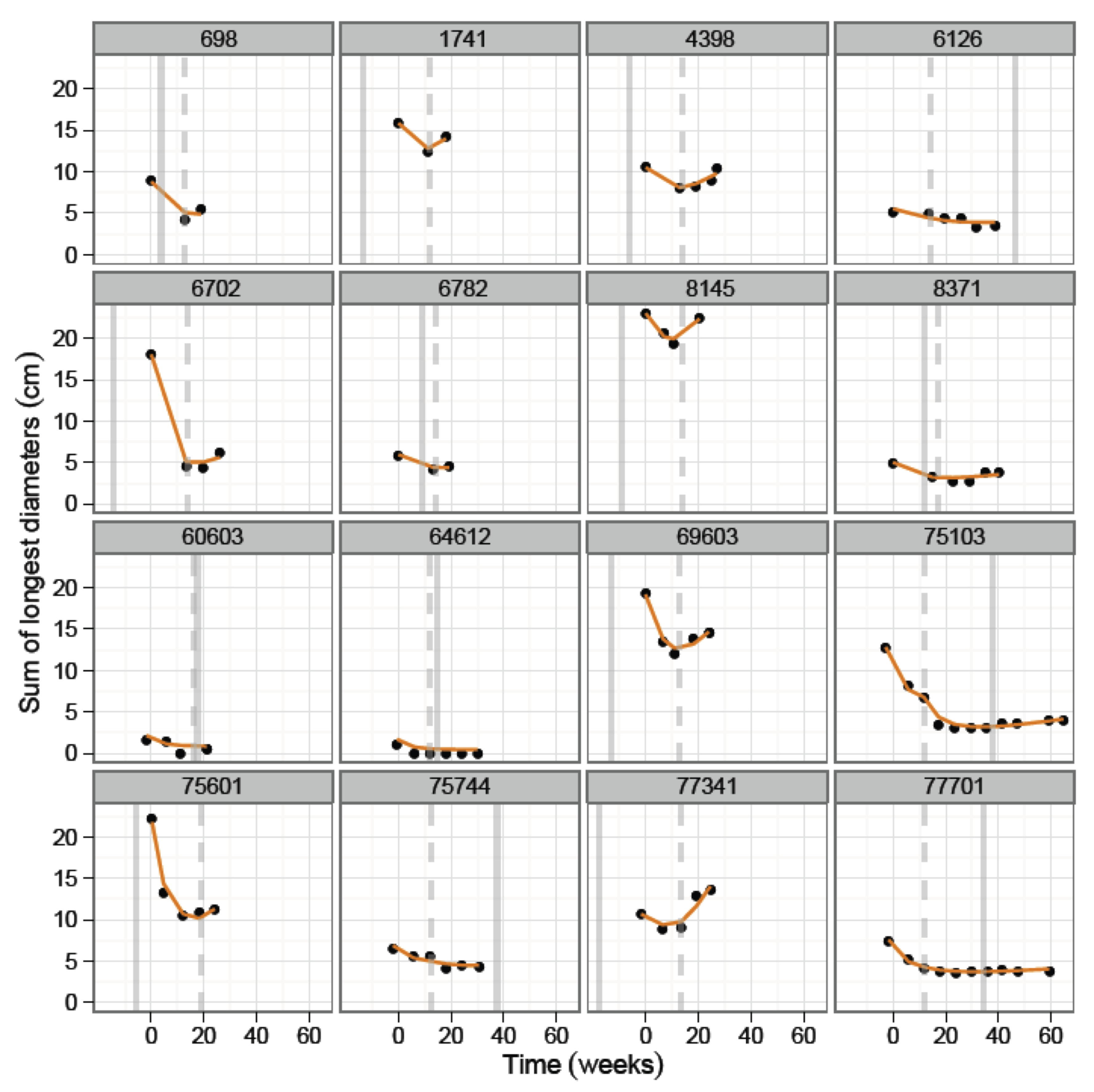

Supplement: Additional file 2: Figure S2. — Model predicted vs. observed tumor size for 16 individuals taken at random. Black solid points: observed tumor size. Orange solid line: model predicted tumor size. Grey dash line: time of randomization (start of maintenance treatment). Grey solid line: estimated time to tumor regrowth (TTG). (TIFF 3041 kb) [file 12885_2016_2455_MOESM2_ESM.tiff]
